# The PolS-PolR Two-Component System Regulates Genes Involved in Poly-P Metabolism and Phosphate Transport in *Microlunatus phosphovorus*

**DOI:** 10.3389/fmicb.2019.02127

**Published:** 2019-09-13

**Authors:** Chuanqing Zhong, Peipei Zhang, Cheng Liu, Meng Liu, Wenbing Chen, Jiafang Fu, Xiaoyu Qi, Guangxiang Cao

**Affiliations:** ^1^School of Municipal and Environmental Engineering, Shandong Jianzhu University, Jinan, China; ^2^Shandong Medicinal Biotechnology Center, Shandong Academy of Medical Sciences, Shandong First Medical University, Jinan, China; ^3^Key Laboratory for Biotech-Drugs of National Health Commission, Jinan, China; ^4^State Key Laboratory of Microbial Technology, School of Life Science, Shandong University, Qingdao, China

**Keywords:** polyphosphate metabolism, two-component system, gene regulation, *Microlunatus phosphovorus*, phosphate transport

## Abstract

*Microlunatus phosphovorus* NM-1 is a polyphosphate (poly-P)-accumulating bacterium that accumulates poly-P under aerobic conditions and degrades poly-P under anaerobic conditions. In this study, the two-component system (TCS) PolS-PolR was identified in NM-1, and the response regulator PolR was found to directly bind to the promoters of genes related to phosphate transport (MLP_RS00235, MLP_RS23035, and MLP_RS24590); poly-P catabolism (MLP_RS12905) and poly-P synthesis (MLP_RS23025). RT-qPCR assays showed that *ppgk* (MLP_RS12905), ppk (MLP_RS23025), *pstS* (MLP_RS23035), and *pit* (MLP_RS24590) were down-regulated during the aerobic-anaerobic shift. The sequence GTTCACnnnnnGTTCaC was identified as a recognition sequence for PolR by MEME analysis and DNase I footprinting. EMSAs and ChIP-qPCR assays indicated that PolR binds to the promoters of *pit* (MLP_RS00235), *ppgk* (MLP_RS12905), *ppk* (MLP_RS23025), *pstS* (MLP_RS23035) and *pit* (MLP_RS24590), and ChIP-qPCR further suggested that the binding affinity of PolR was lower under anaerobic conditions than under aerobic conditions *in vivo*. These findings indicate that the PolS-PolR TCS in *M. phosphovorus* may be involved in the regulation of poly-P metabolism in response to levels of dissolved oxygen in the environment, and our results provide insights into new approaches for understanding the mechanisms of phosphorus accumulation and release.

## Introduction

High concentrations of phosphorus are a leading factor in surface water eutrophication, and eutrophication may occur when the total phosphorus amount achieves 200–500 mg/m^2^/year in the water (Strokal et al., 2014). However, the residual phosphorus in waste-water can be efficiently removed by an activated sludge process, known as the enhanced biological phosphorus removal (EBPR) system, which contains cyclic anaerobic and aerobic stages (Winkler et al., 2011; Wang et al., 2015; Zhang et al., 2015). The EBPR process produces an effluent with a very low phosphorus content and a sludge enriched in phosphorus, from which microorganisms can “extravagantly” take up more inorganic phosphate than needed metabolically and convert the soluble phosphate to the insoluble phase in the form of intracellular polyphosphate (poly-P) (Tarayre et al., 2016).

The microorganisms that contributed to enriching poly-P in the EBPR system were called polyphosphate-accumulating organisms (PAOs) (Mino, 2000). *Acinetobacter spp*. were first investigated as PAOs, and “*Candidatus* Accumulibacter,” *Lampropedia sp., Tetrasphaera elongate*, and *Microlunatus phosphovorus* were also considered potentially responsible for phosphorus-removal in activated sludge (Crocetti et al., 2000; He and McMahon, 2011; Tarayre et al., 2016; Coats et al., 2017). *M. phosphovorus* NM-1 (*Actinobacteria, Propionibacteriaceae*), which was isolated and identified in 1995, can accumulate large amounts of poly-P (Nakamura et al., 1995), and this species accounts for 9.0% of PAOs in the EBPR system (Mino, 2000; Coats et al., 2017).

The genome sequence of *M. phosphovorus* NM-1 was reported in 2012 (Kawakoshi et al., 2012). Dozens of genes were predicted to be involved in the metabolic processing of poly-P, including: *ppk2* (MLP_RS02760, MLP_RS24205) and *ppk* (MLP_RS23025) which encode polyphosphate kinases (PPK) that catalyze the conversion of ATP into poly-P; *ppx* (MLP_RS21635), which encodes the exopolyphosphatase (PPX) that subsequently hydrolyzes the terminal residues of poly-P into inorganic phosphate (Pi); *ppgk* (MLP_RS02595, MLP_RS12905) encoding the polyphosphate glucokinase (PPGK) that phosphorylates glucose using poly-P; *pap* (MLP_RS11265), encoding a polyphosphate:AMP phosphotransferase (PAP) that synthesizes ADP using poly-P and AMP; and *ppnk* (MLP_RS08475), which encodes a polyphosphate/ATP-dependent NAD kinase (PPNK) that synthesizes NADP+ using poly-P and ATP. In addition, *pstSCAB* (MLP_RS23035, MLP_RS23040, MLP_RS23045, and MLP_RS23050) encode the high-affinity Pi-specific transport (Pst) system PstSCAB, which can transport phosphate into cells under low phosphorus concentrations. *pit* (MLP_RS00235, MLP_RS14415, and MLP_RS24590) encode three low-affinity inorganic Pi transport (Pit) systems, which can transport inorganic phosphate into or out of the cell under high phosphorus concentrations (Tanaka et al., 2003; He and McMahon, 2011; Kawakoshi et al., 2012; Tu and Schuler, 2013). However, the mechanisms regulating poly-P biosynthesis have not been fully delineated, and there are no reports about the global regulation of poly-P metabolism in *M. phosphovorus*.

Previous studies revealed that PAOs, including *M. phosphovorus*, can synthesize poly-P under aerobic conditions and degrade poly-P under anaerobic conditions (Tsai and Liu, 2002; Coats et al., 2017), although the regulatory mechanism remained unclear. Two-component systems (TCSs), containing a response regulator (RR) and histidine kinase (HK), normally respond to changes in environmental factors and influence development and metabolism in bacteria (Zschiedrich et al., 2016). In this study, we identified the TCS PolS-PolR, which is annotated as MLP_RS10490—MLP_RS10500 in *M. phosphovorus* NM-1 and which may be involved in the regulation of poly-P metabolism in response to the level of dissolved oxygen. Target genes of PolS-PolR that are related to poly-P metabolism were also detected in *M. phosphovorus* NM-1, and our results provide insights into new approaches for investigating mechanisms of phosphorus accumulation and release.

## Materials and Methods

### Strains and Culture Conditions

*Microlunatus phosphovorus* NM-1 was obtained from the American Type Culture Collection (Accession number ATCC700054) and grown in a Sequencing Batch Reactor (SBR) containing synthetic waste-water as described previously (Zhong et al., 2018a). *Escherichia coli* strains DH5α and BL21(DE3)pLysS were used, respectively, for general cloning and for the expression of PolR. All *E. coli* strains were cultured in Luria-Bertani (LB) medium or on LB-agar medium, and antibiotics were added as needed.

### Heterologous Expression and Purification of His-Tagged PolR

The *polR* gene of *M. phosphovorus* NM-1 was amplified using primers PolR-28aFor and PolR-28aRev (Table S1) and then inserted into the pMD18-T vector. After confirmation by DNA sequencing, *polR* gene was cloned into pET-28a resulting in the pET-PolR recombinant vector, which was then introduced into *E. coli* BL21(DE3)pLysS for protein expression. His-tagged PolR protein was induced by isopropyl β-D-1-thiogalactopyranoside and purified using Ni-NTA. The polyclonal antibody to PolR in rabbits was produced by GenScript Biotech Corporation (Nanjing, China).

### Western Blotting Assay

**Figure 1:**
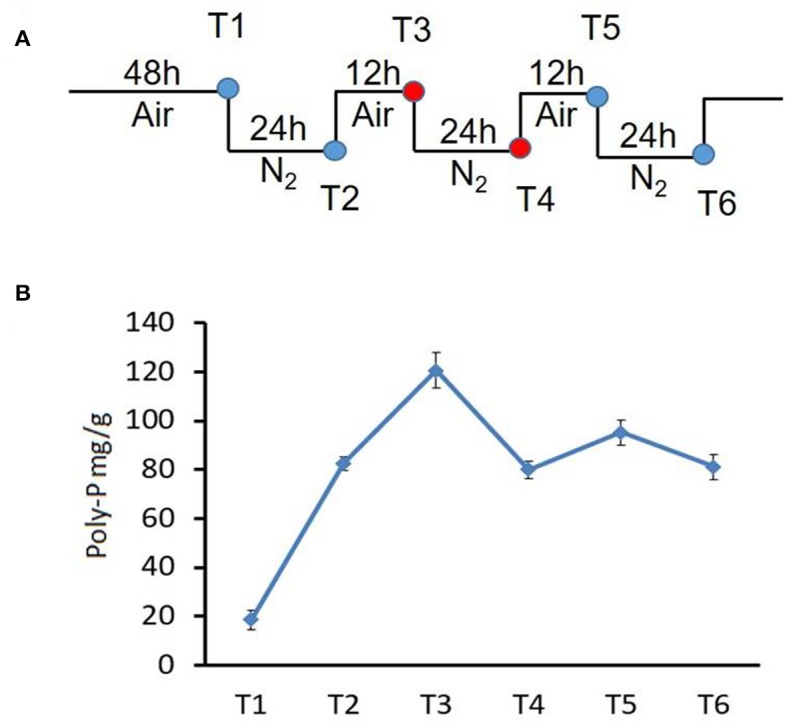
Changes in poly-P content during growth of strain NM-1 in the SBR system. **(A)** Diagram of the aerobic-anaerobic shifts during the SBR process. T1–T6 refer to growth stages. **(B)** Cell pellets were collected from the SBR at different times, and the poly-P content was determined. The values were obtained by three independent experiments and are shown as means ± standard deviations.

To evaluate the expression of PolR in NM-1, cells from culture stages T3 and T4 in the SBR (Figure 1A) were collected by centrifugation at 10,000 g for 10 min, and then pellets were washed and resuspended in PBS buffer followed by disruption with the sample preparation system (FastPrep, MP). Protein samples in the supernatants were obtained by centrifugation and then separated using a NuPAGE 4–12% gradient polyacrylamide gel (Invitrogen). The protein samples were transferred onto Hybond ECL membrane (GE Life Sciences) by a Trans-blot device (Bio-Rad). The polyclonal antibody to PolR and the Western ECL detection system (Amersham) were used in the immunoblotting assay.

### Poly-P Content Analysis

To investigate the metabolism of poly-P under aerobic and anaerobic conditions, bacterial cells were collected at stages T1-T6 from the SBR (Figure 1A), and the amounts of poly-P were determined using described methods (Zhong et al., 2018a).

### Electrophoretic Mobility Shift Assays (EMSAs)

The fragments of promoters of genes involved in poly-P metabolism were amplified by PCR using primers listed in Table S1. The PCR products were labeled by biotin at the 3′-end using the Biotin 3′ End DNA Labeling kit (Thermo Scientific). Purified His-tagged PolR was used in the assays, with described procedures (Zhang et al., 2014). For competition assays, excess of unlabelled specific probes and non-specific probes were added into the reaction. The mixtures were analyzed on non-denaturing page (8% polyacrylamide), then the DNA probes were transferred to nylon membrane and developed by ECL Western blotting Analysis System (GE Healthcare).

### RNA Extraction and Real-Time Quantitative PCR (RT-qPCR)

For total RNA extraction, bacterial cells were collected by centrifugation at stages T3–T4 from the SBR, and then the cells were disrupted using the FastPrep sample preparation system. TRIzol (Takara) was used for the extraction of total RNA, and Turbo DNA-free DNase reagents (Thermofisher) were used to remove DNA contamination. RNA samples were then reverse transcribed into cDNA using an M-MLV reverse transcriptase kit (Takara). RT-qPCR was performed by using SYBR Premix Ex Taq kit (TaKaRa) on Roche LightCycler480 thermal cycler. According to the recommended conditions of kit, the relative amounts of cDNA were normalized for the quantity of 16s rRNA. The primers used for RT-qPCR are listed in Table S1.

### DNase I Footprinting Assay

DNase I footprinting was performed as described (Wang et al., 2012). Briefly, the promoter fragment of *pit* (MLP_RS00235) was amplified and ligated into the pMD18-T vector, and then the DNA fragment was amplified from the resulting plasmid template using primers M13F-47 (FAM-labeled) and M13R-48, and purified as a probe. A certain amount of probes were incubated with 0 μg PolR and 3.0 μg PolR for 30 min at room temperature, followed by incubated with DNase I for 1 min at 25°C. And then the reactions were stopped by DNase I stop solution and extracted by phenol/chloroform. DNA samples were precipitated by ethanol and dissolved in ultrapure water.

### Chromatin Immunoprecipitation and Real-Time Quantitative PCR Assay (ChIP-qPCR Assay)

The chromatin immunoprecipitation assay was performed as previously described (Fu et al., 2019). Bacterial cells were collected at stages T3 and T4 from the SBR, and the polyclonal antibody to PolR and protein G-Agarose (Sigma-Aldrich) were used to recover target DNA. A qPCR assay was performed to determine the enrichment of promoter fragments of genes related to poly-P metabolism (primers are listed in Table S1). For the negative control, the input DNA (non-immunoprecipitated) was used as template.

### Identification of a PolR DNA Binding Motif

The sequences of promoters bound by PolR were used as input for the MEME software tool (Bailey et al., 2015) and RegPredict (Novichkov et al., 2010) for the motif search.

## Results

### *M. phosphovorus* NM-1 Accumulates Poly-P Under Aerobic Conditions and Degrades Poly-P During Anaerobic Conditions

To investigate poly-P metabolism in *M. phosphovorus* NM-1, the strain was cultured in synthetic waste-water in an SBR. Strain NM-1 was first incubated under aerobic conditions for 48 h (T1), and the air was then replaced by N_2_ to maintain anaerobic conditions for 24 h (T2), followed by another aerobic phase for 12 h (T3), and then a second anaerobic phase for 24 h (T4) (Figure 1A). A third aerobic phase was then carried out for 12 h (T5), followed by a third anaerobic phase for 24 h (T6) (Figure 1A). The poly-P content of strain NM-1 was analyzed during the SBR stages, reaching 18.61 mg/g cells at T1 and 82.64 mg/g cells at T2. Poly-P increased to 120.58 mg/g in the second aerobic phase (T3) but decreased to 80.02 mg/g in the following anaerobic phase (T4). Poly-P again increased in the third aerobic phase, to 95.21 mg/g at T5, followed by another decrease in the third anaerobic phase, to 81.12 mg/g at T6 (Figure 1B). These changes in poly-P content indicated that strain NM-1 can synthesize poly-P under aerobic conditions and degrade poly-P during anaerobic conditions, which is consistent with previously reported results (Nakamura et al., 1995).

### PolS-PolR Is Predicted to Be a TCS in *M. phosphovorus* NM-1

**Figure 2:**
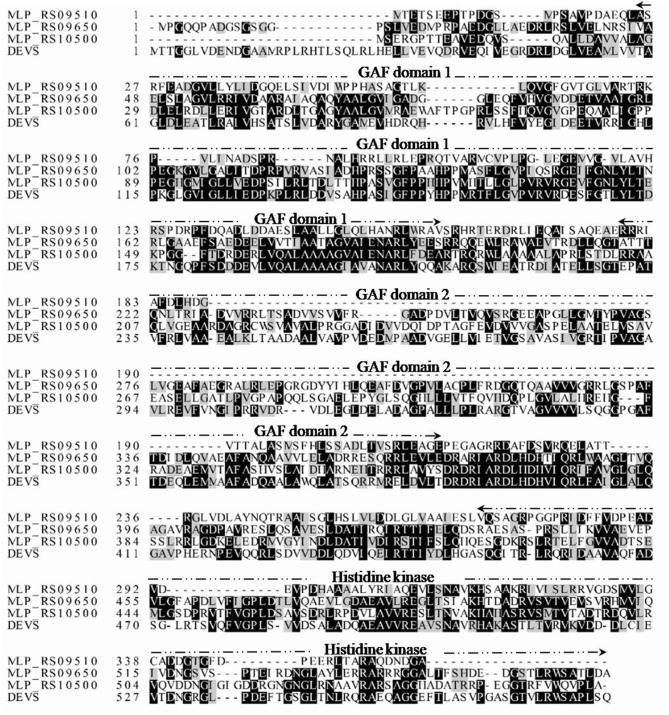
Multiple sequence alignment of *M. tuberculosis* DevS with putative GAF-containing proteins of strain NM-1. Darkened background indicates conserved amino acids. GAF domain 1, GAF domain 2, and the histidine kinase domain are marked by dotted lines.

Although the metabolism of poly-P differed in NM-1 under aerobic and anaerobic conditions, the genes regulating the response to the changing levels of dissolved oxygen remained largely uncharacterized. In *Mycobacterium tuberculosis*, the DevS-DevR TCS responds to oxygen levels, with the GAF domain of the HK DevS sensing oxygen signals, and following phosphorylation by DevS, the RR DevR regulates the expression of dormancy-related genes (Madrona et al., 2016; Lobao et al., 2019). We speculated that a TCS might mediate the transition response to changing oxygen levels in *M. phosphovorus*, and bioinformatics analysis indicated that its genome (NC_015635.1) contained three genes encoding proteins with GAF-like domains: MLP_RS09510, MLP_RS09650, and MLP_RS10500 (Figure 2). MLP_RS09510 has low similarity with DevS, and MLP_RS09650 is an orphan histidine kinase. MLP_RS10500 has 38% amino acid identity with DevS, and similar to DevS, it contains two GAF domains at the N terminus and a histidine kinase-like ATPase at the C terminus. The second GAF domain of DevS was demonstrated to sense signals such as oxygen, hypoxia, CO or NO (Madrona et al., 2016). In addition, MLP_RS10490 has 76% identity with DevR, which has a helix-turn-helix (HTH) DNA binding domain. Given their close location on the genome and their similarities to DevS and DevR, we predicted that MLP_RS10500 and MLP_RS10490 formed a TCS involved in the hypoxic signal transduction pathway, and we named this TCS PolS-PolR for the putative regulation of poly-P metabolism.

### PolR Binds to the Promoters of Five Genes Related to Poly-P Metabolism *in vitro*

**Figure 3:**
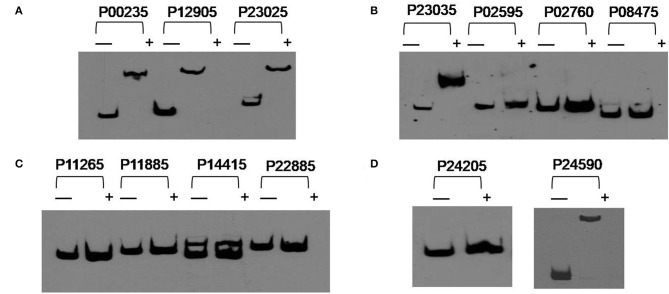
PolR binding to the promoter regions of predicted target genes. **(A–D)** EMSAs of the promoter regions of predicted target genes with purified His-tagged PolR. Probes for different promoters were incubated either without protein (–) or with 3.0 μg PolR (+). Poly(dI-dC) (1.0 μg) was added to all samples as competitor.

In order to investigate if the genes related to poly-P metabolism are targets of PolR (MLP_RS10490), EMSAs were performed using His-tagged PolR protein. The promoters of genes involved in the synthesis and hydrolysis of poly-P were amplified by PCR and labeled by biotin for use as probes. The results indicated that PolR binds to the promoters of *pit* (MLP_RS00235, MLP_RS24590), *ppgk* (MLP_RS12905), *ppk* (MLP_RS23025), and *pstS* (MLP_RS23035) (Figure 3), which is consistent with a previous report (Zhong et al., 2018b), in which EMSA shows the homologous protein of PolR binds to the promoters of responding genes in *M. phosphovorus* JN459, suggesting that these genes could be targets of PolR. The results of competitive EMSAs assay (Figure S1) indicated that PolR binds to the promoters specifically. *ppgk* (MLP_RS12905), which encodes polyphosphate glucokinase, is responsible for the catabolism of poly-P. *pstS* (MLP_RS23035) encodes a component of the PstSCAB transport system, which is involved in the transport of phosphate into cells at low environmental phosphorus levels, whereas *pit* (MLP_RS00235) and *pit* (MLP_RS24590) encode a Pit transport system for transporting phosphate at high phosphate levels. *ppk* (MLP_RS23025) encodes a polyphosphate kinase that is involved in the synthesis of poly-P. We hypothesized that PolR could regulate poly-P metabolism by directly regulating the transcription of genes related to phosphate uptake and synthesis or catabolism of poly-P. While PolR could not bind to the promoters of *ppgk* (MLP_RS02595), *ppk2* (MLP_RS02760, MLP_RS24205), *ppnk* (MLP_RS08475), *pap* (MLP_RS11265), *phoB* (MLP_RS11885), *pit* (MLP_RS14415), and *phoU* (MLP_RS22885) (Figure 3), suggesting that these genes are not directly regulated by PolR.

### Expression of Several PolR Target Genes Changed Significantly During the Aerobic-Anaerobic Shift

**Figure 4:**
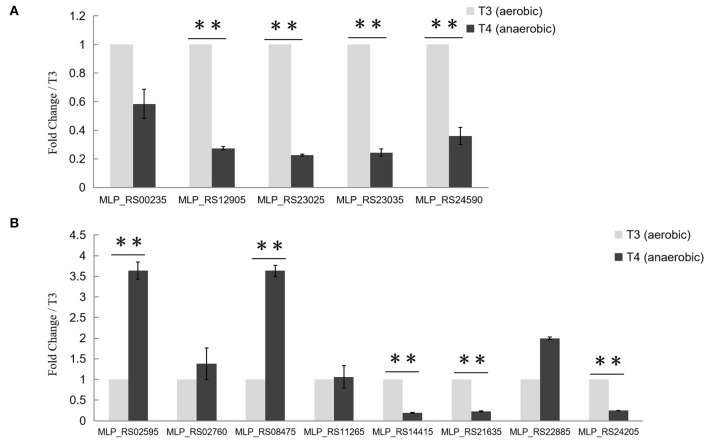
Transcriptional analysis of Poly-P metabolism related genes. **(A)** Transcriptional analysis of putative target genes of PolR. The expression of *pit* (MLP_RS00235, MLP_RS24590), *ppgk* (MLP_RS12905), *ppk* (MLP_RS23025), and *pstS* (MLP_RS23035) at stage T3 (aerobic condition, light gray) and stage T4 (anaerobic condition, dark gray) was determined by RT-qPCR. **(B)** Transcriptional analysis of other Poly-P metabolism related genes. The transcription levels of genes at T3 were set to one, and the expression of the 16S rRNA gene was used as the control. The values were obtained by three independent experiments and are shown as means ± standard deviations. ^**^, compared with the expression data at stage T3, *P* < 0.01.

To find out whether the transcription of target genes of PolR was changed during the aerobic-anaerobic shift, the transcription levels of *pit* (MLP_RS00235, MLP_RS24590), *ppgk* (MLP_RS12905), *ppk* (MLP_RS23025), and *pstS* (MLP_RS23035) in *M. phosphovorus* NM-1 at T3 (aerobic) and T4 (anaerobic) were analyzed by RT-qPCR. Expression of *ppgk* (MLP_RS12905), *ppk* (MLP_RS23025), *pstS* (MLP_RS23035), and *pit* (MLP_RS24590) was significantly down-regulated under anaerobic conditions compared to aerobic conditions (Figure 4A). The transcription levels of *ppgk* (MLP_RS12905), *ppk* (MLP_RS23025), *pstS* (MLP_RS23035), and *pit* (MLP_RS24590) were 3.7-, 4.1-, 4.2-. and 2.8-fold higher at T3 than at T4, respectively. However, the transcription of *pit* (MLP_RS00235) showed no significant change (Figure 4A).

The transcription levels of other genes related to poly-P metabolism were also analyzed in this study (Figure 4B). *ppgk* (MLP_RS02595), *ppnk* (MLP_RS08475), *ppx* (MLP_RS21635), and *pit* (MLP_RS14415) which are related to the catabolism of poly-P, exhibited lower expression levels at stage T3 than at stage T4, whereas *ppk2* (MLP_RS24205), the gene responsible for poly-P synthesis, was up-regulated at T3. While the expression levels of *ppk2* (MLP_RS02760), *pap* (MLP_RS11265), and *phoU* (MLP_RS22885) at T3 showed no obvious difference with T4. A similar transcription pattern was found in *M. phosphovorus* JN459, as determined by RNA-seq assay (Table S2). Strain JN459, with a little higher concentration of Poly-P than that of NM-1, was derived from strain NM-1 through spontaneous mutation in EBPR system, while the mechanism of high ability to accumulate Poly-P remains unclear (Zhong et al., 2018a).

### A Consensus Sequence for PolR Binding in Target Promoters

**Figure 5:**
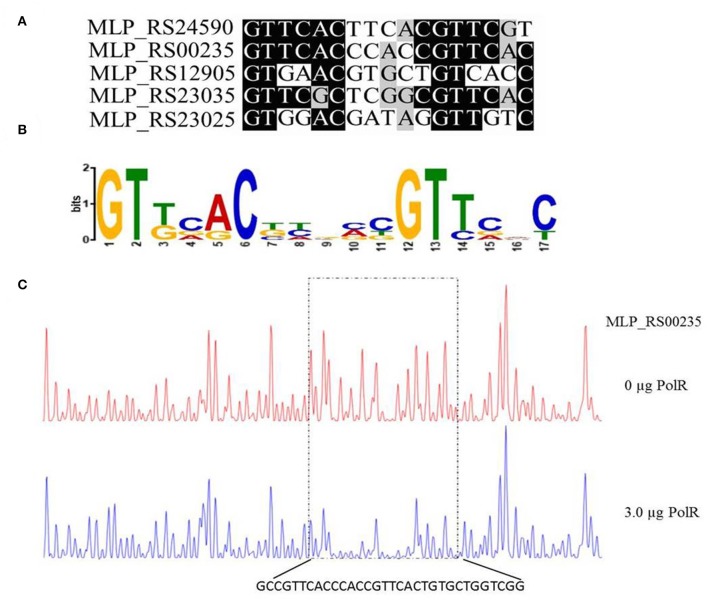
Determination of the PolR DNA binding sequence. **(A)** Alignment of promoters of target genes of PolR. The conserved nucleotides are marked by a dark background. **(B)** The consensus DNA binding site of PolR predicted by RegPredict. **(C)** PolR-protected region upstream of *pit* (MLP_RS00235) determined by DNaseI footprinting. The control reaction (no protein) is shown in red. The reaction including 3.0 μg PolR is shown in blue. The dotted box indicates the protected region. The nucleotides protected by PolR are shown beneath the chromatograms.

Based on the promoter targets of PolR, we deduced a conserved DNA binding sequence for PolR by MEME analysis (Figure 5A). The 17-bp consensus sequence GTTCACnnnnnGTTCaC, containing 6-bp direct repeats, was found in all five promoters bound by PolR *in vitro* (Figure 5B), whereas this sequence was not found in the promoters of genes that were not targets of PolR in the EMSAs. In order to validate the sequence as a target site for PolR binding, a DNase I footprinting assay was performed. A 32-bp region from the *pit* (MLP_RS00235) promoter was protected by PolR, and this region included the deduced consensus sequence (Figure 5C), providing further evidence that GTTCACnnnnnGTTCaC is the recognition sequence for PolR binding.

### PolR Binding to Target Promoters *in vivo* Differs Under Aerobic and Anaerobic Conditions

**Figure 6:**
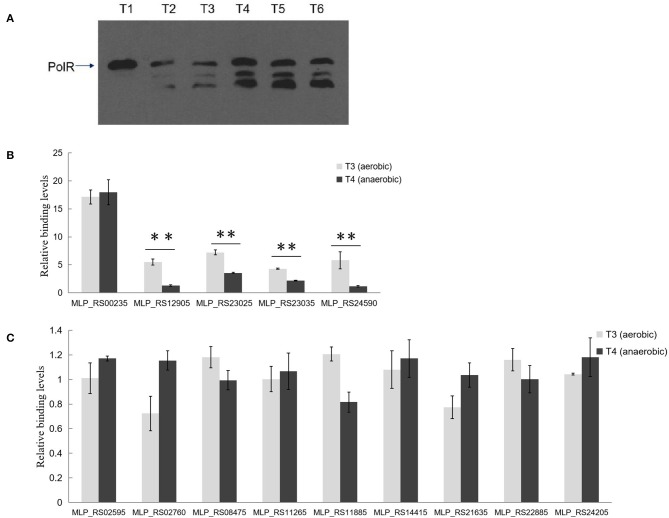
PolR binds to the promoter of genes associated with poly-P metabolism and phosphate transport *in vivo*. **(A)** The expression of PolR during growth in the SBR system was detected by Western blotting assay using anti-PolR. **(B)** The enrichment of the promoters of *pit* (MLP_RS00235, MLP_RS24590), *ppgk* (MLP_RS12905), *ppk* (MLP_RS23025), and *pstS* (MLP_RS23035) at stage T3 (aerobic condition, light gray) and stage T4 (anaerobic condition, dark gray) was analyzed by ChIP-qPCR assay. **(C)** The enrichment of the promoters of other Poly-P metabolism related genes. The relative enrichment value of each gene was normalized for the 16S rRNA gene, and the enrichment value of each gene in the input DNA samples was arbitrarily set to one. The values were obtained by three independent experiments and are shown as means ± standard deviations. ^**^, compared with the data at stage T3, *P* < 0.01.

Western blotting analysis with anti-PolR revealed bands corresponding to PolR protein in lysates of strain NM-1 and indicated that PolR is expressed under both aerobic and anaerobic conditions (Figure 6A). Two additional bands responding to the antibody of PolR were detected at T2–T6 stage, and we speculate that these bands might be the different modification of PolR. To confirm the target genes of PolR *in vivo*, a ChIP assay was performed with anti-PolR and strain NM-1 growing at stages T3 and T4, and qPCR was then performed to detect recovery of promoter fragments of genes associated with poly-P metabolism. The results showed that a region upstream of *pit* (MLP_RS00235) was enriched at T3 (17.13 ± 2.21) and T4 (17.97 ± 3.88) (Figure 6B), suggesting that PolR binds to the promoter of *pit* (MLP_RS00235) under both aerobic and anaerobic conditions. The promoter of *ppgk* (MLP_RS12905) was enriched at T3 (5.50 ± 0.98), but no enrichment was detected at T4 (Figure 6B). The promoter fragment of *ppk* (MLP_RS23025) was more highly enriched at T3 (7.23 ± 0.44) than at T4 (3.56 ± 0.11) (Figure 6B), suggesting that the binding affinity of PolR for the promoter of *ppk* (MLP_RS23025) under aerobic conditions is stronger than under anaerobic conditions. Similarly, the enrichment of the *pstS* (MLP_RS23035) promoter at T3 (4.32 ± 0.08) was about 2-fold higher than at T4 (2.16 ± 0.07), and the *pit* (MLP_RS24590) promoter was also more highly enriched at T3 (5.83 ± 1.53) than at T4 (1.17 ± 0.12) (Figure 6B). The ChIP-qPCR assay also showed that the promoters of the other genes associated with poly-P metabolism were not significantly enriched in the ChIP sample compared to the input sample, which is consistent with the EMSAs (Figure 6C).

## Discussion

The biosynthesis and degradation pattern of poly-P in PAOs is usually consistent with the hypothesis that the bacteria catabolize poly-P to supply energy for growth under anaerobic conditions (Zhou et al., 2010). However, the global regulatory mechanisms controlling poly-P metabolism in PAOs remained unclear, including for *M. phosphovorus*. Our findings in this study suggested that the PolS-PolR TCS may be involved in poly-P metabolism and soluble phosphorus transport. PolS has 38% amino acid identity to DevS, the HK that responds to hypoxia in *M. tuberculosis* (Sousa et al., 2017), suggesting that the PolS-PolR TCS may also respond to the levels of dissolved oxygen.

**Figure 7:**
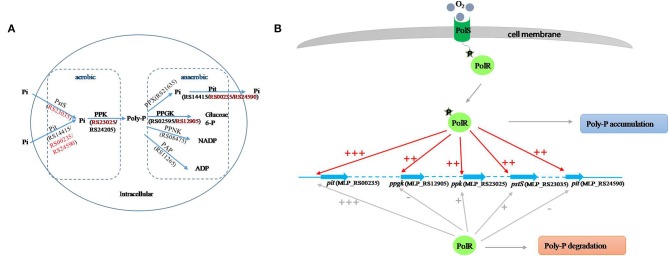
Proposed model of the PolS-PolR regulatory role in poly-P metabolism and Pi transport. **(A)** The diagram of poly-P metabolic pathway in *M. phosphovorus* NM-1 (He and McMahon, 2011; Kawakoshi et al., 2012). The locus tags and gene symbols related to the metabolism of Poly-P were annotated. The target genes of PolR were marked in red. **(B)** The putative mechanism of PolR regulating poly-P metabolism and Pi transport. The regulation of target genes by non-phosphorylated and phosphorylated PolR is indicated by gray lines and red lines, respectively. The relative degree of promoter binding is indicated by the number of “+,”and “–” indicates that PolR does not bind to the promoter.

Poly-P is the signature metabolite produced by *M. phosphovorus* under aerobic conditions, and 11 genes were predicted to be involved in the metabolic processing of poly-P, including three phosphate permeases in the Pit system and a gene cluster for the phosphate transport system PstSCAB. Our study showed notable changes in the transcription levels of most of the genes involved in poly-P metabolism and Pi transport during the aerobic-anaerobic shift, with results similar to those found for strain JN459 (Zhong et al., 2018a). The transcription levels of the four genes *ppgk* (MLP_RS12905), *ppk* (MLP_RS23025), *pstS* (MLP_RS23035) and *pit* (MLP_RS24590) were down-regulated significantly at T4, which is an anaerobic stage, compared to levels at the aerobic stage T3. Additionally, these four genes were confirmed as targets of PolR by EMSAs and the ChIP-qPCR assay, and the binding affinities of PolR to their promoters *in vivo* were lower under anaerobic conditions than aerobic conditions. Combined with the transcriptional changes, we deduce that PolR positively regulates the transcription of the four target genes under aerobic conditions and that this control is reduced under anaerobic conditions. In the ChIP-qPCR assay, the high enrichment was detected at the promoter of *pit* (MLP_RS00235), which encodes the Pit system associated with phosphate transport under high phosphate levels. However, no obvious difference in enrichment was detected for this promoter between aerobic and anaerobic conditions, suggesting that PolR positively regulates the expression of *pit* (MLP_RS00235) under both aerobic and anaerobic conditions for phosphorus retention in cells (Figure 7).

In *M. tuberculosis*, the HK DevS/DosT are activated when the GAF domain senses hypoxia signals or oxygen, resulting in phosphorylation of DevR, which then binds to the promoters of the dormancy regulon and positively regulates the transcription of the associated genes. On the other hand, although GAF domains of DevS and DosT share 75% amino-acid sequence identity, they sense different signals (Kumar et al., 2007; Kaur et al., 2016; Lobao et al., 2019). Moreover, protein All4978 from Cyanobacterium *Nostoc* sp. PCC7120, containing GAF domain and a DNA binding motif, binds DNA preferentially when exposed to hypoxia (Tang et al., 2015). It is deduced that GAF domain could sense the different signals and work in different ways. In addition, FNR (*E. coli K12*) and WhiB3 (*M. tuberculosis*) both sense oxygen/NO by an iron-sulfur cluster [[4Fe−4S]]. While oxygen/NO inhibit the regulatory function of FNR but active that of WhiB3 (Green et al., 2014), suggesting that it has a possibility that regulators with similar sensing domain work in different ways. In this study, the binding of PolR to the *ppgk* (MLP_RS12905), *ppk* (MLP_RS23025), *pstS* (MLP_RS23035), and *pit* (MLP_RS24590) promoters declined in the aerobic-anaerobic shift, suggesting that hypoxic conditions repressed the phosphorylation of PolS and consequently reduced activation of PolR. We hypothesize that oxygen may be the signal sensed by PolS, although this hypothesis requires further study (Figure 7B).

Under aerobic growth conditions, the anabolism of poly-P is greater than its catabolism in *M. phosphovorus*, resulting in poly-P accumulation, which is opposite to the pattern of poly-P metabolism in strains cultured under anaerobic conditions. There are three *ppk* genes encoding PPK in the NM-1 strain, and these genes are responsible for the synthesis of poly-P from ATP (He and McMahon, 2011; Kawakoshi et al., 2012). According to our results with RT-qPCR (Lobao et al., 2019), genes related to the biosynthesis of poly-P, including *ppk* (MLP_RS23025) and *ppk2* (MLP_RS24205), were significantly down-regulated under anaerobic conditions, while the transcription level of *ppk2* (MLP_RS02760) showed no obvious changes under aerobic or anaerobic conditions (Figure 4B). PPNK (MLP_RS08475), PAP (MLP_RS11265), PPX (MLP_RS21635), and PPGK (MLP_RS02595, MLP_RS12905) are the enzymes that catalyze poly-P into NADP, ADP, Pi and glucose-6-phosphate, respectively (He and McMahon, 2011; Kawakoshi et al., 2012). Therefore, it is surprising that *ppx* (MLP_RS21635) and *ppgk* (MLP_RS12905), which are responsible for poly-P catabolism, were down-regulated under anaerobic conditions, whereas *ppnk* (MLP_RS08475) and *ppgk* (MLP_RS02595) were up-regulated (Figure 4B). We speculate that when strains are exposed to aerobic conditions, *ppk* (MLP_RS23025), and *ppk2* (MLP_RS02760, MLP_RS24205) are involved in the synthesis of poly-P while *ppx* (MLP_RS21635) and *ppgk* (MLP_RS12905) mainly participate in poly-P degradation. When the growth conditions shift to an anaerobic stage, *ppk* (MLP_RS23025) and *ppk2* (MLP_RS24205) are the major genes involved in poly-P biosynthesis, and *ppnk* (MLP_RS08475) and *ppgk* (MLP_RS02595) are the major genes involved in poly-P degradation. Of these genes, the PolS-PolR TCS identified in this study directly regulates *ppk* (MLP_RS23025) and *ppgk* (MLP_RS12905) and thereby contributes to regulating poly-P metabolism during the aerobic-anaerobic shift.

Except for poly-P accumulation in aerobic condition, PHA synthesis in anaerobic condition was considered as an important trait of PAOs (Mino, 2000). While in *M. phosphovorus*, PHA was produced in aerobic condition which is different from that in proteobacterial PAOs (Akar et al., 2006). PHA was likely to be synthesized by β-oxidation pathway and independent to the poly-P accumulation (Kawakoshi et al., 2012). The results of RNA-seq assay indicated that the transcription level of MLP_RS06230 which could be related to the PHA production was significantly down-regulated under anaerobic conditions compared to aerobic conditions in *M. phosphovorus* JN459 (Kawakoshi et al., 2012; Zhong et al., 2018a). It is deduced that the expression of MLP_RS06230 is also relevant to the dissolved oxygen level. While no conserved DNA binding sequence for PolR or similar sequence was found in the promoter of MLP_RS06230 based on the sequence alignment, thus we speculate that the PHA production is not directly regulated by PolR but other TCS or regulators.

The PhoB-PhoR TCS regulates phosphate transport in *E*. *coli*; PhoB is a positive regulator, which is activated by PhoR under low Pi concentrations and which induces the expression of the PstSCAB system and inhibits the Pit system (Lamarche et al., 2008; Lubin et al., 2016). PhoP in *Streptomyces*, the homologous protein of PhoB, actives the transcription of genes related to scavenging, transport of phosphate under phosphate depletion conditions (Martin et al., 2017). Our study indicated that the binding sites of PolR are highly similar with that of PhoP indentified in *Streptomyces* which is composed of direct repeat units of 11 nucleotides GtTCAccnnnn (Sola-Landa et al., 2008; Martin et al., 2017). A PhoB-PhoR TCS, encoded by MLP_RS11885 and MLP_RS11890, also exists in *M. phosphovorus*, suggesting that the PhoB-PhoR TCS could recognize the promoters of target genes of PolR and regulate phosphate transport and phosphate metabolism. It is very likely that PolR regulates the expressions of target genes responsed to the oxygen concentration while PhoP regulates the expressions of target genes responsed to the phosphate concentration. In addition, the MLP_RS22875-MLP_RS22880 TCS of *M. phosphovorus* is homologous to the SenX3-RegX3 TCS of *M. tuberculosis*, which regulates the PPK-encoding gene Rv2984 and thereby affects the synthesis of poly-P (Sanyal et al., 2013), suggesting that the expression of *ppk* may also be regulated by the MLP_RS22875-MLP_RS22880 TCS. We therefore anticipate that poly-P metabolism in strain NM-1 may be co-regulated by PolS-PolR, PhoB-PhoR and SenX3-RegX3.

In conclusion, *M. phosphovorus* strain NM-1 accumulates poly-P under aerobic conditions and degrades poly-P under anaerobic conditions. In this study, we identified the PolS-PolR TCS (MLP_RS10500 and MLP_RS10490), which shows high similarity with DevS-DevR of *M. tuberculosis*. We further demonstrated that PolR binds *in vitro* to the promoters of genes associated with phosphate metabolism, including *pit* (MLP_RS00235. MLP_RS24590), *ppgk* (MLP_RS12905), *ppk* (MLP_RS23025), and *pstS* (MLP_RS23035), and ChIP-qPCR revealed that PolR also binds to these promoters *in vivo* under aerobic conditions and suggested that the binding affinity was lower under anaerobic conditions. Transcriptional analyses showed that four of these genes, *ppgk* (MLP_RS12905), *ppk* (MLP_RS23025), *pstS* (MLP_RS23035), and *pit* (MLP_RS24590), were notably down-regulated when strains were cultivated under anaerobic conditions following aerobic growth. We also identified the consensus sequence GTTCACnnnnnGTTCaC as the conserved binding site of PolR. We speculate that PolS senses an oxygen signal and activates PolR, which directly induces the transcription of the poly-P catabolic gene *ppgk* (MLP_RS12905); the poly-P synthesis gene *ppk* (MLP_RS23025); the phosphate transport genes *pit* (MLP_RS00235, MLP_RS24590), and *pstS* (MLP_RS23035) to influence the metabolism of poly-P and phosphate transport.

## Data Availability

All datasets generated for this study are included in the manuscript/Supplementary Files.

## Author Contributions

GC designed the experiment. CZ performed the experiment. PZ prepared the manuscipt. CL and ML analyzed the data. WC, JF, and XQ revised the article.

### Conflict of Interest Statement

The authors declare that the research was conducted in the absence of any commercial or financial relationships that could be construed as a potential conflict of interest.

## Supplementary Material

Figure S1 Specific binding of PolR to the promoter regions of target genes. The labelled promoters were incubated with 0 μg (lane 1) PolR, 1.0 μg (lane 2) PolR and 3.0 μg (lane 3-5) PolR. 150-fold excess of unlabelled specific probes (lane 4) and nonspecific probes (lane 5) were added in the reactions. Poly(dI-dC) (1.0 μg) was added to all samples as competitor.

Table S1 Primers used in this study.

Table S2 Information of genes related to Poly-P metabolism and phosphorus transport.
