# Supplementary figures and images for: The PolS-PolR Two-Component System Regulates Genes Involved in Poly-P Metabolism and Phosphate Transport in Microlunatus phosphovorus
Source: Front Microbiol. 2019 Sep 13;10:2127. doi: 10.3389/fmicb.2019.02127 (PMC6754071; doi:10.3389/fmicb.2019.02127)

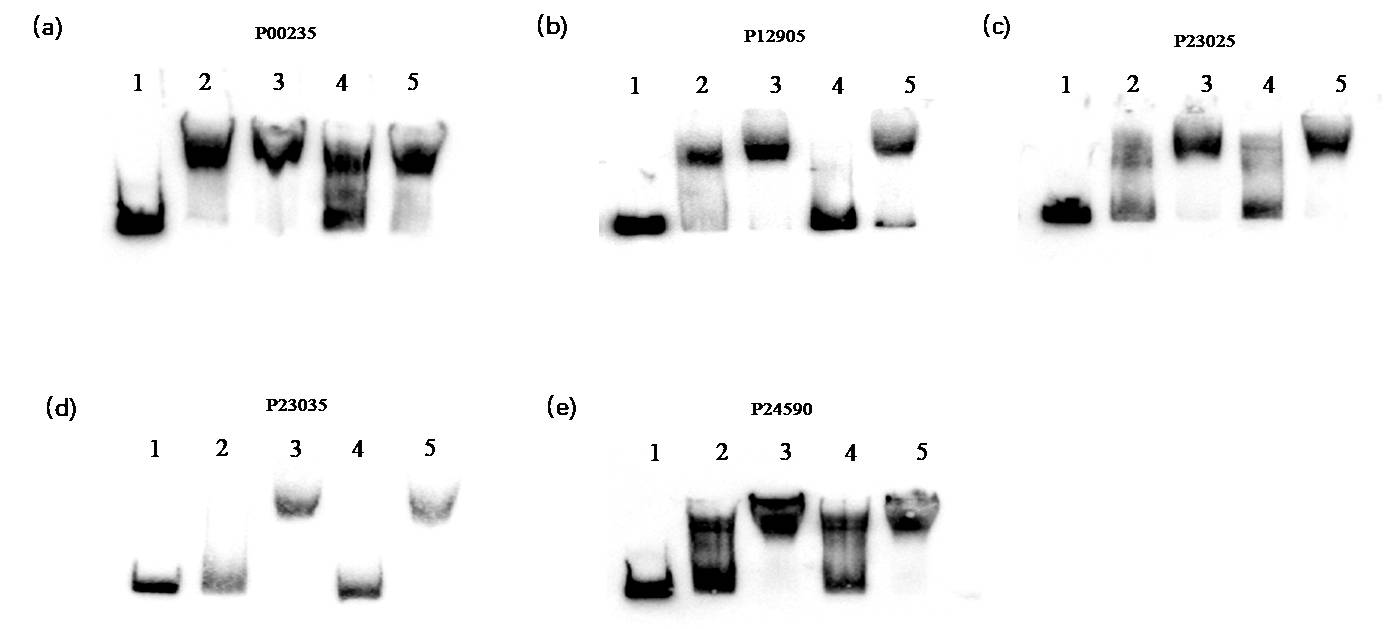

Supplement: Figure S1 — Specific binding of PolR to the promoter regions of target genes. The labelled promoters were incubated with 0 μg (lane 1) PolR, 1.0 μg (lane 2) PolR and 3.0 μg (lane 3-5) PolR. 150-fold excess of unlabelled specific probes (lane 4) and nonspecific probes (lane 5) were added in the reactions. Poly(dI-dC) (1.0 μg) was added to all samples as competitor. [file Image_1.JPEG]
