# Supplementary material for: The PolS-PolR Two-Component System Regulates Genes Involved in Poly-P Metabolism and Phosphate Transport in Microlunatus phosphovorus
Source: Front Microbiol. 2019 Sep 13;10:2127. doi: 10.3389/fmicb.2019.02127 (PMC6754071; doi:10.3389/fmicb.2019.02127)
Supplement: Table S1 — Primers used in this study. [file Table_1.DOC]

**Supplementary material**

Table S1 Primers used in this study

| **Oligonucleotides** | **DNA Sequence （5’→3’）** |
| --- | --- |
| **For recombinant plasmid pET28a-PolR construction** | |
| PolR-28aFor | CCCATATGAGCGCGTCCCCCGGTGAGGTCGGCCGC* |
| PolR-28aRev | CCGAATTCTCAACGCAGCAGCTTGGCGGCCAACAC |
| **For EMSAs** | |
| P00235For | cgaccagcacagtgaacggt |
| P00235Rev | gacagcgatcacggagatca |
| P02595For | tgtgctccaccgtacgctga |
| P02595Rev | atttgcagcactccgttcgc |
| P02760For | tgccgggcgaacaatgggtt |
| P02760 Rev | ttcgacctccgtattcggct |
| P08475For | gaggaatcgaagggggcggg |
| P08475Rev | gaccatctcgccaccttcgc |
| P11265For | tcgctctcggtgttgatcga |
| P11265Rev | cacgcggaccctcgaccatc |
| P12905For | ggcagggtgaacgtgctgtc |
| P12905Rev | tgtgtcccttccgtgaaacc |
| P14415For | tcgtctcggcagcccttggg |
| P14415Rev | gagcttcggtcacgcgacca |
| P22885For | cagttcacagtcgcgatact |
| P22885Rev | ggattcctcgggattgatca |
| P23025For | ctaggagtgtgctgatcccc |
| P23025Rev | tcagccatctggacaaccta |
| P23035For | agacgagtgctgccgtgcac |
| P23035Rev | atcttcactcgttccccttg |
| P24205For | cggtccagagtttatccatc |
| P24205Rev | gagaggcccaaggtgagcaa |
| P24590For | ctttgacagaaccactggct |
| P24590Rev | cagcacagtcacgtagaggt |
| **For RT-qPCR** |  |
| RS00235 rt F | cgctggcgctgctgttcgattt |
| RS00235 rt R | gttcgcaacagcggtcccgacg |
| RS02595 rt F | tgggtgaccaccgccgggatg |
| RS02595 rt R | tggccaccggattgttcgccg |
| RS02760 rt F | cgcgtcgcggccctcgaa |
| RS02760 rt R | cgggcggcgatccggtcg |
| RS08475 rt F | gccgtcgagaccgccgcc |
| RS08475 rt R | tccacgccgcgagcctgg |
| RS11265 rt F | caggcgcaccagcccatcctga |
| RS11265 rt R | cggcaaccgcaggacctcgctc |
| RS12905 rt F | cggttccggcttgacaccccg |
| RS12905 rt R | atccggatgcaggttggccgc |
| RS14415 rt F | ccggaaacgcgatggccacc |
| RS14415 rt R | cgatcgggacgccgctgctg |
| RS21635 rt F | gcgcgcagttgctcgccg |
| RS21635 rt R | cgccgaggctcgggtcga |
| RS22885 rt F | aggtggcgatcgccgtccga |
| RS22885 rt R | ggcgaccggggactgccgag |
| RS23025 rt F | cggccgcttcctggaccgg |
| RS23025 rt R | ccggcgacccggaccatga |
| RS23035 rt F | ccgccgctgtcttggcgatg |
| RS23035 rt R | tggaaggccgcgacccaggt |
| RS23040 rt F | ccgagcgactggcgttcatccc |
| RS23040 rt R | gccgcctcctcatgcagcttcg |
| RS24205 rt F | ggccgtgatgcggcgggc |
| RS24205 rt R | cgatttcgcccgccgcgg |
| RS24590 rt F | ccaggacgatctcgggcggc |
| RS24590 rt R | atcacggcgccgatcaggcc |
| 16s rRNA rt F | gttgtaaaccgctttcag |
| 16s rRNA rt R | tatcggaagcaggctcag |
| **For ChIP-qPCR** |  |
| P00235 ChIP F | tggagtggcggcgaaac |
| P00235 ChIP R | cgtgtttgctccctcctc |
| P02595 ChIP F | tgtgctccaccgtacgctga |
| P02595 ChIP R | atttgcagcactccgttcgc |
| P02760 ChIP F | gcagtcacggacagcatc |
| P02760 ChIP R | acctccgtattcggctatt |
| P08475 ChIP F | gaggaatcgaagggggcggg |
| P08475 ChIP R | gaccatctcgccaccttcgc |
| P11265 ChIP F | tcgctctcggtgttgatcga |
| P11265 ChIP R | cacgcggaccctcgaccatc |
| P12905 ChIP F | ggcgacctgtcttgcctgag |
| P12905 ChIP R | cgagaatccgagccgctt |
| P14415 ChIP F | tcgtctcggcagcccttggg |
| P14415 ChIP R | gagcttcggtcacgcgacca |
| P21635 ChIP F | acttgccacgtcgaactgcc |
| P21635 ChIP R | tcgcgcgagtgaactctccg |
| P22885 ChIP F | cagttcacagtcgcgatact |
| P22885 ChIP R | ggattcctcgggattgatca |
| P23025 ChIP F | ctaggagtgtgctgatcccc |
| P23025 ChIP R | tcagccatctggacaaccta |
| P23035 ChIP F | agacgagtgctgccgtgcac |
| P23035 ChIP R | atcttcactcgttccccttg |
| P23040 ChIP F | tgtcgaccgatgtcgaggct |
| P23040 ChIP R | tgcggctccacgtcagaggg |
| P24205 ChIP F | cggtccagagtttatccatc |
| P24205 ChIP R | gagaggcccaaggtgagcaa |
| P24590 ChIP F | acggctttgacagaacca |
| P24590 ChIP R | gggaatgtcggtgtcaac |
| **For DNase I footprinting** |  |
| P00235For | cgaccagcacagtgaacggt |
| P00235Rev | gacagcgatcacggagatca |

* The restriction enzyme sites are underlined and were used for cloning purposes.
