# Supplementary material for: The PolS-PolR Two-Component System Regulates Genes Involved in Poly-P Metabolism and Phosphate Transport in Microlunatus phosphovorus
Source: Front Microbiol. 2019 Sep 13;10:2127. doi: 10.3389/fmicb.2019.02127 (PMC6754071; doi:10.3389/fmicb.2019.02127)
Supplement: Table S2 — Information of genes related to Poly-P metabolism and phosphorus transport. [file Table_2.DOCX]

**Supplementary material**

**Table S2 Information of genes related to Poly-P metabolism and phosphorus transport.**

| New locus tag | Old locus tag | Gene  symbol | Expression changes/aerobic stage in NM-1 | Expression changes/aerobic stage in JN459(Zhong et al., 2018a.) | PolR binding  In vitro | PolR binding  In vivo (ChIP-T3/  Input) | PolR binding  In vivo (ChIP-T4/  Input) |
| --- | --- | --- | --- | --- | --- | --- | --- |
| MLP_RS00235 | MLP_00530 | *pit* | 0.58 | 1.78 | + | 17.13 | 17.97 |
| MLP_RS02595 | MLP_05430 | *ppgk* | 3.64 | 1.87 | - | 1.01 | 1.17 |
| MLP_RS02760 | MLP_05750 | *ppk2* | 1.38 | 0.48 | - | 0.72 | 1.15 |
| MLP_RS08475 | MLP_17420 | *ppnk* | 3.63 | 1.77 | - | 1.18 | 0.99 |
| MLP_RS11265 | MLP_23310 | *pap* | 1.06 | 0.65 | - | 1.00 | 1.07 |
| MLP_RS11885 | MLP_24520 | *phoB* | 1.23 | 1.79 | - | 1.21 | 0.82 |
| MLP_RS12905 | MLP_26610 | *ppgk* | 0.27 | 0.04 | + | 5.50 | 1.31 |
| MLP_RS14415 | MLP_29830 | *pit* | 0.19 | 0.05 | - | 1.08 | 1.17 |
| MLP_RS22885 | MLP_47360 | *phoU* | 2.00 | 0.88 | - | 1.16 | 1.00 |
| MLP_RS23025 | MLP_47700 | *ppk* | 0.23 | 0.37 | + | 7.23 | 3.56 |
| MLP_RS23035 | MLP_47720 | *pstS* | 0.24 | 0.14 | + | 4.32 | 2.16 |
| MLP_RS24205 | MLP_50300 | *ppk2* | 0.25 | 0.27 | - | 1.04 | 1.18 |
| MLP_RS24590 | MLP_51060 | *pit* | 0.36 | 0.13 | + | 5.83 | 1.17 |

- T
